# Digital Twin-Driven Intrusion Detection for Industrial SCADA: A Cyber-Physical Case Study

**DOI:** 10.3390/s25164963

**Published:** 2025-08-11

**Authors:** Ali Sayghe

**Affiliations:** Department of Electrical Engineering, Yanbu Industrial College, Yanbu 46452, Saudi Arabia; sayghea@rcjy.edu.sa

**Keywords:** digital twin, scada security, cyber-physical systems, anomaly detection

## Abstract

The convergence of operational technology (OT) and information technology (IT) in industrial environments, such as water treatment plants, has significantly increased the attack surface of Supervisory Control and Data Acquisition (SCADA) systems. Traditional intrusion detection systems (IDS), which focus solely on network traffic, often fail to detect stealthy, process-level attacks. This paper proposes a Digital Twin-driven Intrusion Detection (DT-ID) framework that integrates high-fidelity process simulation, real-time sensor modeling, adversarial attack injection, and hybrid anomaly detection using both physical residuals and machine learning. We evaluate the DT-ID framework using a simulated water treatment plant environment, testing against false data injection (FDI), denial-of-service (DoS), and command injection attacks. The system achieves a detection F1-score of 96.3%, a false positive rate below 2.5%, and an average detection latency under 500 ms, demonstrating substantial improvement over conventional rule-based and physics-only IDS in identifying stealthy anomalies. Our findings highlight the potential of cyber-physical digital twins to enhance SCADA security in critical infrastructure. In the following sections, we present the motivation and approach underlying this framework.

## 1. Introduction

Industrial Control Systems (ICS) and Supervisory Control and Data Acquisition (SCADA) networks are central to the operation of critical infrastructure in sectors such as water, energy, and oil and gas. While these systems were originally deployed in isolated, air-gapped environments with minimal cybersecurity, most ICS and SCADA installations now face increased cyber risk due to the integration of operational technology (OT) with information technology (IT), enabled by the Industrial Internet of Things (IIoT), remote monitoring, and data-driven optimization [1].

This growing connectivity introduces new vectors for sophisticated cyber-physical threats. High-profile incidents such as Stuxnet, Industroyer, and Triton illustrate how attackers can manipulate control logic, falsify sensor readings, or disrupt communication protocols, causing significant real-world damage [2,3,4].

Most current intrusion detection systems (IDS) in industrial networks are adapted from IT security practices, relying on signature-based methods or network anomaly detection [5,6,7]. However, these solutions often fail to detect process-aware threats where attackers subtly manipulate sensor readings or actuator commands to disrupt operations while keeping network traffic within expected norms, thereby evading traditional alarms [8,9].

Recent research has begun to explore process-aware and hybrid IDS approaches, including those based on Digital Twins (DTs), virtual replicas that simulate physical systems using process models and real-time data [10,11,12,13,14,15]. Despite this progress, existing DT-based solutions still struggle to reliably detect stealthy attacks that subtly alter process states.

In summary, this paper offers several novel contributions beyond the current state-of-the-art in ICS security. (1) We introduce a digital twin-driven IDS for SCADA that tightly integrates high-fidelity physics simulation with live SCADA network traffic via OPC UA, enabling process-aware detection not achievable by prior DT-based IDS [13,14]. (2) We develop an adversarial attack simulation toolkit to inject both cyber (network) and physical process attacks, providing a rigorous and repeatable evaluation environment. (3) We propose a hybrid anomaly detection engine that fuses physics-based residual analysis and machine learning (LSTM for network anomalies, one-class SVM for process anomalies) in a unified framework. (4) We present extensive experimental results against stealthy, multi-stage attacks on a 72 h SCADA dataset, demonstrating improved detection over traditional IDS baselines. This fusion strategy detects subtle, simultaneous deviations across the cyber-physical domain that single-modality detectors (either physics-only or ML-only) often miss, addressing a key gap identified in prior studies [14,16,17]. (5) We present an extensive experimental evaluation against stealthy, multi-stage attacks on a 72 h SCADA dataset. The results (detection F1-score ∼96%, with low false alarms and sub-second latency) demonstrate significantly improved detection performance over traditional IDS baselines (e.g., Snort and purely physics-based detectors), clearly validating the advantages of our hybrid DT-ID approach. Unlike earlier DT-based IDS frameworks, which were often tested only on straightforward attack scenarios or in simulation, our work uniquely shows that a physics–ML fused system can reliably uncover even stealthy false-data attacks that maintain “normal-looking” behavior. These contributions position the proposed DT-ID system as a comprehensive advancement beyond prior work in securing industrial control systems.

The remainder of this paper is structured as follows. Section 2 reviews related work in SCADA security, Digital Twin applications, and ML-based intrusion detection. Section 3 presents the architecture of the proposed DT-ID framework. Section 4 describes the case study setup and experimental methodology. Section 5 discusses the results and system performance. Section 6 concludes the paper and outlines future work.

## 2. Related Work

Industrial control systems (ICS) and Supervisory Control and Data Acquisition (SCADA) networks have traditionally relied on intrusion detection systems (IDS) derived from information technology (IT) environments. Signature-based and network anomaly detection tools such as Snort [5] and Suricata [6] have long served as primary defenses in industrial networks. While effective at detecting known attack signatures and obvious network anomalies, these methods generally lack awareness of the physical processes they protect, limiting their effectiveness against stealthy, process-level attacks that manipulate sensor values or control logic without generating conspicuous network events [8,9,18]. Network monitors like Bro (now Zeek) [7] have improved protocol inspection granularity but still primarily operate at the packet or flow level rather than at the process level.

To overcome these limitations, the research community has increasingly turned to process-aware and hybrid intrusion detection frameworks. Early surveys by Mitchell and Chen [18] and Giraldo et al. [8] emphasize the importance of integrating cyber-physical context into IDS, highlighting how attacks exploiting the physical process layer can evade purely network-based monitoring. Residual-based detection approaches, which use model-based or data-driven methods to estimate expected sensor values and flag deviations as potential attacks, have shown promise. However, these methods remain vulnerable to stealthy attacks that mimic the statistical properties of normal operation, as shown in recent adversarial studies [9,10,11,12,13,14,15].

The emergence of Digital Twin (DT) technology has provided new opportunities to improve ICS and SCADA security. DTs are high-fidelity, real-time virtual models of physical systems capable of synchronizing with live data streams to simulate, monitor, and optimize process behavior. Oyekan and Hu [11] demonstrated a DT-based cybersecurity monitoring framework for pipeline systems, showing the potential for real-time state comparison and anomaly detection. Zhang et al. [12] expanded on this by integrating DTs with process analytics for smart manufacturing. Recent studies, such as Zhao et al. [13] and Lin et al. [14], have leveraged cloud-based DT platforms and edge analytics for scalable anomaly detection in industrial IoT environments. Xu et al. [15] further employed generative adversarial networks (GANs) to enhance the robustness of DT-based anomaly detection in water treatment plants, achieving higher sensitivity to certain classes of attacks. However, many DT-driven solutions still focus on anomaly detection in simplified settings and may not generalize well to adversarial scenarios involving carefully crafted false data injection (FDI) or zero-day threats.

Hybrid intrusion detection systems that combine machine learning (ML) with process models have also gained momentum. Pan et al. [16] introduced a hybrid deep learning and physics-guided DT architecture for detecting cyber-physical anomalies, demonstrating strong results on benchmark datasets. Xu et al. [17] proposed a fusion approach combining DT representations with deep learning to detect zero-day attacks in smart manufacturing systems. Nonetheless, adversarial machine learning remains a persistent challenge in ICS security. Creswell et al. [19] and related studies have shown that GAN-based attacks can generate process-consistent yet malicious sensor data, successfully bypassing even advanced hybrid detectors. This raises concerns about the resilience of current ML-based and hybrid IDS in the face of adaptive, stealthy adversaries.

In addition, a review of recent industry incident reports [1] and benchmark datasets such as SWaT [20], WADI [21], and BATADAL [22] indicate that most experimental validations remain confined to testbed or simulated environments. Real-world deployments introduce further complexity, including variable process dynamics, sensor drift, incomplete observability, and evolving adversarial tactics. While some frameworks offer limited retraining or adaptive learning, continuous model updating to address new threats remains an open problem.

Recent digital twin plus machine learning approaches for ICS intrusion detection illustrate the community’s progress toward more holistic defenses, yet important gaps remain. For example, Zhao et al. [13] and Lin et al. [14] both leverage digital twins in combination with cloud-based analytics or deep learning models to detect anomalies in industrial IoT and SCADA environments. While these systems achieve good detection rates, they primarily focus on either network attacks or simplified process models, and they do not incorporate an integrated attack simulation environment or real-time control response. In contrast, our DT-ID framework blends a high-fidelity process twin with local ML analysis and a response module, all evaluated under live adversarial conditions. Xu et al. [15] introduced a GAN-enhanced DT for anomaly detection in a water treatment testbed, demonstrating improved sensitivity to certain attacks. However, their approach emphasizes offline analysis (detecting anomalies in logged data) and targets a narrow set of attacks, whereas our work performs online detection and covers a broader spectrum of threats, including adaptive and zero-day scenarios. Similarly, recent hybrid methods by Pan et al. [16] and Xu et al. [17] combine physics-based models with deep learning to detect ICS attacks (even aiming at zero-days). These studies report high accuracy on standard datasets (often exceeding 90% detection rates), but they generally assume the availability of well-labeled attack data or focus on pre-recorded incidents. Our proposed system advances beyond these SOTA efforts by (i) providing a real-time, closed-loop detection capability (with the digital twin continuously synchronized with live process data at 100 Hz), (ii) actively challenging the IDS with adversarially crafted stealthy attacks to ensure robustness (most prior works do not evaluate on such adaptive threats), and (iii) integrating an automated response mechanism, which many academic DT+ML IDS prototypes lack. In summary, compared to existing systems, the DT-ID framework offers a more comprehensive and adaptive defense strategy, fusing multiple detection paradigms and validating them under conditions that closely mimic real-world advanced persistent threats.

## 3. Core Components

**Figure 1:**
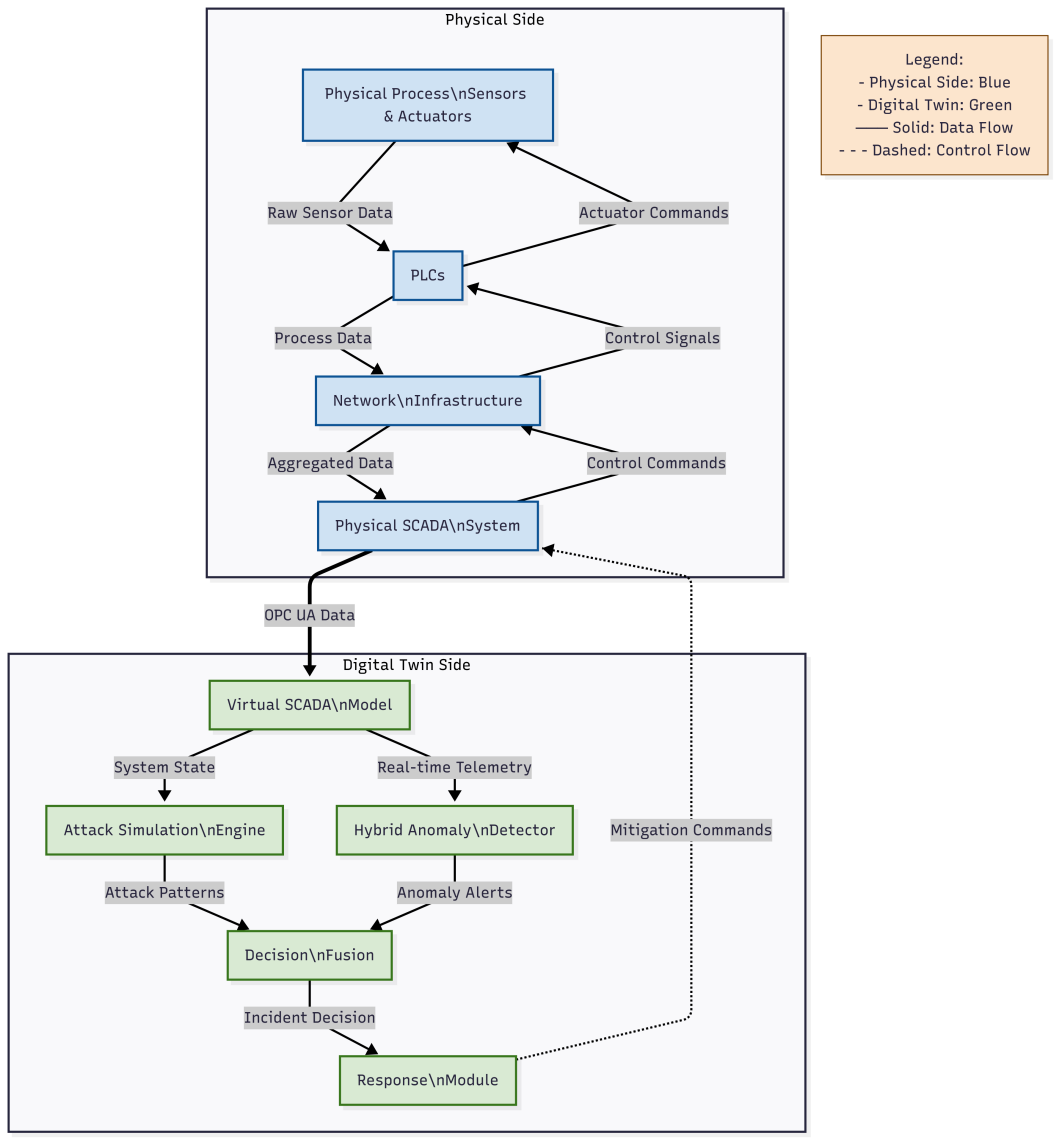
DT-ID system architecture: integration of virtual SCADA, attack simulation, hybrid detection modules, and response coordination.

The architecture of the proposed DT-ID system is shown in Figure 1. The system is composed of four tightly integrated modules: a high-fidelity virtual SCADA model (Digital Twin), an adversarial attack simulation engine, a hybrid anomaly detector, and a multi-stage response module. Together, these modules enable real-time monitoring, robust adversarial testing, adaptive anomaly detection, and automated mitigation in industrial SCADA environments.

### 3.1. Virtual SCADA Model

The Virtual SCADA Model serves as a high-fidelity digital twin, emulating the core dynamics of a water treatment process, including tank hydraulics and chemical dosing. The digital twin and process simulation were implemented in MATLAB Simulink, with all data handling and synchronization routines executed in Python 3.10. This dual-environment setup enables flexible model development, rapid prototyping, and integration with external attack libraries. The module leverages established physical models to provide process-aware context for anomaly detection and attack simulation.

The hydraulic behavior of each storage tank is modeled by Bernoulli’s principle:

(1) $$\frac{dH}{dt}=\frac{Q_{\mathrm{in}}-\beta \sqrt{H}}{A}$$

where *H* denotes the tank level, $Q_{\mathrm{in}}$ the inflow rate, β the valve coefficient, and *A* the cross-sectional area.

pH control dynamics are captured as:

(2) $$\frac{dpH}{dt}=k\left(C_{\mathrm{acid}}-C_{\mathrm{base}}\right)-\gamma pH$$

where *k* and γ are kinetic parameters, and $C_{\mathrm{acid}}$ and $C_{\mathrm{base}}$ are dosing concentrations.

To maintain alignment with the real or simulated plant, the digital twin performs state synchronization at 100 Hz using a delta-based update. A synchronization frequency of 100 Hz was chosen to ensure that the virtual model remains tightly coupled to real-world or simulated process dynamics, thereby enabling the prompt detection of rapid, stealthy attacks that could otherwise evade slower polling rates. This high update rate is critical for capturing transient anomalies in critical infrastructure. If the absolute difference between the physical and virtual parameters exceeds a specified tolerance, the virtual state is resynchronized.

The tolerance for state resynchronization is set according to sensor resolution and expected physical noise levels (e.g., ±0.5% for tank level sensors), ensuring the digital twin is robust to normal fluctuations while remaining sensitive to anomalous deviations caused by attacks. This process is formalized in Algorithm 1.
**Algorithm 1** Delta-based twin synchronization**Require:** physical state, virtual state, tolerance  1:**for** each parameter *x* in physical state **do**  2:      δ← |physical state[x]−virtual state[x]|  3:      **if** δ> tolerance[x]  **then**  4:            virtual state[x]← physical state[x]  5:      **end if**  6:**end for**

#### Analytical Basis for Delta-Synchronization

To determine a principled resynchronization threshold (ϵ) for each state variable, we analyze the process dynamics equations. For the tank level *H*, Equation (1) can be used to estimate the maximum rate of change. Given the maximum inflow rate ($Q_{\mathrm{in}},\max$) and valve coefficient β, the fastest increase in *H* over a small interval Δt (0.01 s) is:

(3) $$\Delta H\approx \frac{Q_{\mathrm{in}},\max}{A}\cdot \Delta t$$

where *A* is the tank cross-sectional area and outflow $\sqrt{H}$ is limited near typical operating levels. For our system, this yields a ΔH on the order of $10^{-3}$ m per 0.01 s under normal conditions.

Similarly, the pH dynamics (Equation (2)) indicate the maximum drift in pH per time step, based on reaction kinetics *k*, γ and dosing limits. We set the synchronization tolerance ϵ for each variable slightly above these theoretical maxima (plus a margin for sensor noise). For example, tank level readings use ϵ≈0.5% of full scale (about 0.025 m in our setup), which is consistent with both sensor resolution and the ΔH derived from Equation (1). In this way, ϵ is derived from the physical model; deviations smaller than ϵ are attributable to normal process variation or sensor noise, whereas larger discrepancies trigger twin state correction.

This approach mirrors the formal coupling strategies in prior Digital Twin works (e.g., differential-equation-based thresholds as in [11]) and ties our twin synchronization directly to the process’s dynamics and noise characteristics. Furthermore, the chosen 100 Hz synchronization frequency is analytically sufficient to capture the fastest system transients; it is an order of magnitude higher than the inverse of the process time constants, ensuring tight temporal alignment between the twin and the physical plant.

### 3.2. Attack Simulation Engine

The Attack Simulation Engine systematically injects adversarial scenarios into the system to rigorously validate the DT-ID framework. Three representative classes of cyber-physical threats are implemented:

- False Data Injection (FDI): Introduces up to ±20% sensor bias or ramps in tank level and pH readings.
- Denial of Service (DoS): Floods the PLC-HMI channel with $10^{4}$ malformed Modbus packets per second.
- Reconnaissance/Command Injection: Performs automated port scans and injects unauthorized commands, e.g., remote actuator manipulation.

The attack library includes both standard industrial threats (such as typical FDI and DoS patterns) and advanced adversarial scenarios, including stealthy zero-day attacks generated using adversarial techniques. This approach allows evaluation of both signature-based and anomaly-based detection capabilities within the testbed.

The full set of attack types and parameters used in the simulation are summarized in Table 1.

Attack events are scheduled in non-overlapping 10 min intervals, randomized over a 72 h simulation period. The start time, duration, and affected system component for each attack are drawn from a uniform random distribution to prevent bias and more realistically simulate unpredictable adversary behavior.

For each attack, the target system component (e.g., PLC, sensor, HMI) is selected at random from the pool of available devices to increase the diversity and unpredictability of the adversarial evaluation. This process ensures that the detection framework is rigorously challenged by a range of both conventional and novel threat scenarios.

**Figure 2:**
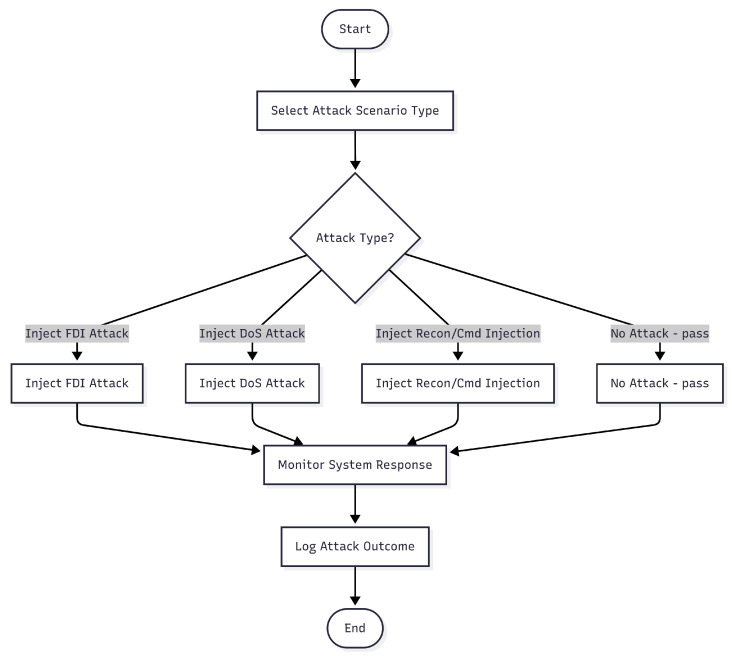
Attack injection workflow: decision branches for each attack type leading to payload execution.

The attack injection logic is detailed in Algorithm 2, and the decision workflow is illustrated in Figure 2.
**Algorithm 2** Attack simulation**Require:** attack type, randomly chosen target device, sensor data  1:**if** attack type = FDI **then**  2:      spoofed ← sensor data[Level1] ×1.2  3:      Inject spoofed Modbus packet to target device  4:**else if** attack type = DoS **then**  5:      Send $10^{4}$ malformed TCP packets to target device  6:**else if** attack type = Recon **then**  7:      Scan target device ports; enumerate Modbus function codes  8:**else**  9:      No attack performed10:**end if**

### 3.3. Hybrid Anomaly Detector

The Hybrid Anomaly Detector is designed to capture both cyber (network-level) and physical (process-level) anomalies in SCADA environments by integrating deep learning and physics-based machine learning.

- Cyber Module:

The cyber module processes sliding windows of Modbus/TCP traffic data using a three-layer Long Short-Term Memory (LSTM) network (each layer with 128 units) augmented by an attention mechanism. Input features include packet sizes, function codes, inter-arrival times, and statistical summaries (mean, variance, and maximum) computed over 60-timestep (one-minute) windows. This module outputs a probability score $P_{\mathrm{cyber}}$ indicating the likelihood of a cyber anomaly.

- Physical Module:

The physical module analyzes process residuals, calculated as $r\left(t\right)=|y_{\mathrm{sensor}}\left(t\right)-y_{\mathrm{DT}}\left(t\right)|$, where $y_{\mathrm{sensor}}\left(t\right)$ is the actual process reading and $y_{\mathrm{DT}}\left(t\right)$ is the digital twin’s prediction. The residual sequence and its derived statistics (mean, standard deviation, and change over rolling 1 s intervals) are provided as input to a one-class Support Vector Machine (SVM) with a radial basis function (RBF) kernel (ν=0.01,γ=0.1). The SVM produces a probability score $P_{\mathrm{physical}}$ for physical anomaly detection.

- Decision Fusion:

The outputs of the cyber and physical modules are combined into a single anomaly score by weighted summation:

(4) $$P_{\mathrm{final}}=0.3\;P_{\mathrm{cyber}}+0.7\;P_{\mathrm{physical}}$$

The fusion weights (0.3 for cyber, 0.7 for physical) were selected via grid search to maximize F1-score on a held-out validation dataset, reflecting the higher reliability of process residuals in this case study. The detection threshold ($P_{\mathrm{final}}>0.85$) was similarly optimized using ROC curve analysis to balance sensitivity and specificity, ensuring low false positive rates while maintaining high detection accuracy.

**Figure 3:**
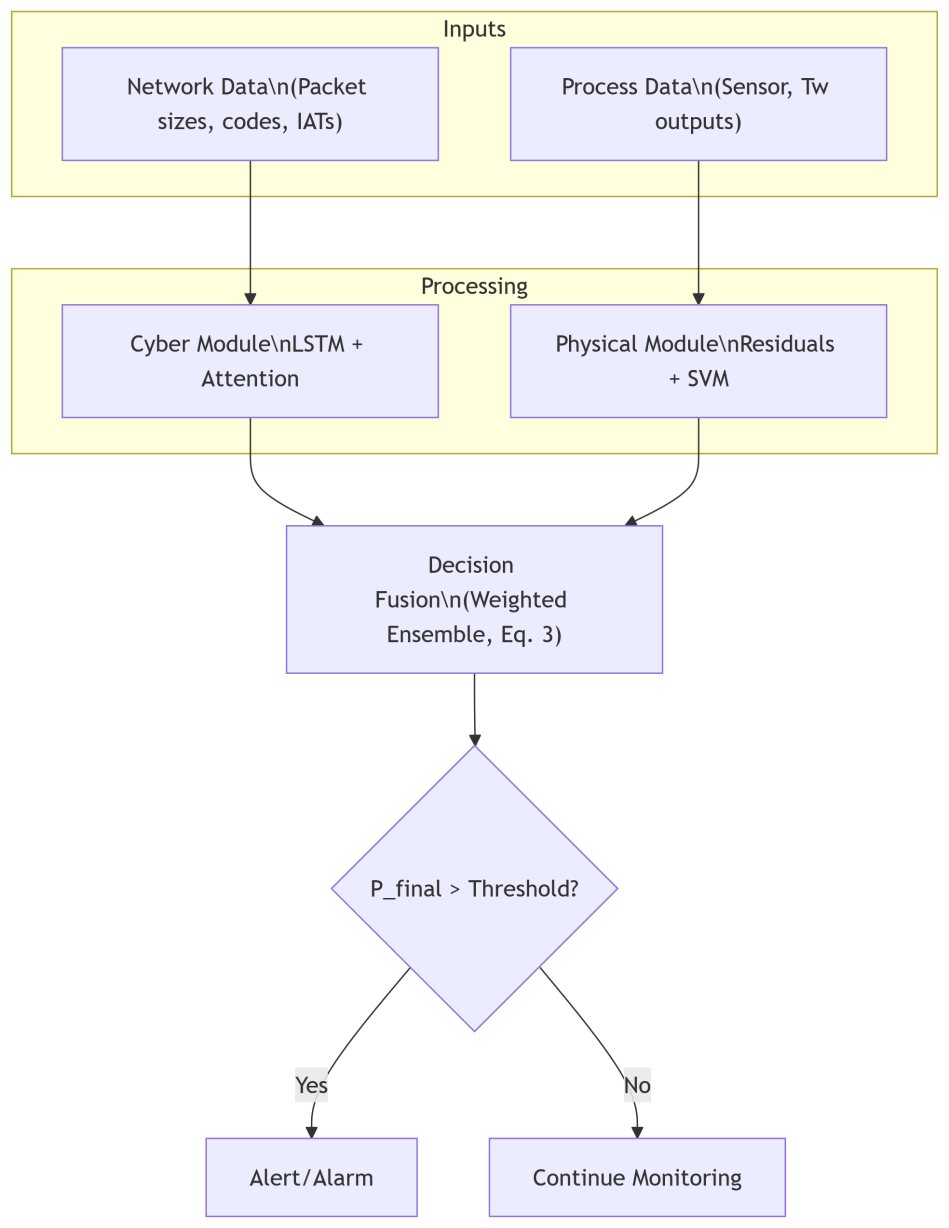
Hybrid anomaly detection workflow: Parallel cyber and physical anomaly detection modules process network traffic and sensor data independently. Their outputs are fused into a unified anomaly score, which is compared against a detection threshold to trigger an alert or continue monitoring. The diagram highlights parallel feature extraction, decision logic, and the formal workflow of the hybrid detection process.

The complete data flow of the hybrid anomaly detector is depicted in Figure 3, illustrating the parallel extraction of cyber and physical features and their integration into a unified alerting mechanism. We selected an LSTM network with an attention mechanism for the cyber anomaly module (instead of, e.g., a CNN) due to its strength in modeling sequential Modbus/TCP patterns over time, and a one-class SVM for the physical module due to its effectiveness in modeling the normal process-state distribution and detecting novel deviations without extensive labeled attack data. This hybrid design was hypothesized to offer superior accuracy by leveraging temporal context and one-class outlier detection.

### 3.4. Response Module

The response module is responsible for initiating mitigation actions upon the detection of a confirmed anomaly. Responses are staged to minimize operational disruption and risk, while also enabling forensic traceability and adaptive system improvement.

Alerting and Human-in-the-Loop:

When an anomaly is detected, the system automatically generates real-time alerts on the operator’s Human Machine Interface (HMI) dashboard. These alerts are accompanied by detailed log entries and can be optionally configured to trigger email or SMS notifications for engineering and security teams. While the system is capable of executing all response actions automatically, it can also be configured for human-in-the-loop operation requiring operator confirmation before critical interventions such as process lockdown or controller fail-over.

Mitigation Actions:

The mitigation process comprises three escalation stages:

- **Stage 1:** The system raises visual and audible alarms on the HMI, while logging all relevant forensic data (such as network packet captures and process sensor snapshots) for post-incident analysis.
- **Stage 2:** If the anomaly persists or is classified as critical, the system enforces PLC command lockdown by restricting process control to a predefined whitelist of safe operations, preventing further unauthorized manipulation.
- **Stage 3:** In the event of sustained or high-severity attack, the system can automatically trigger fail over to redundant backup controllers to maintain process continuity and safety.

Adaptive Learning and Concept Drift:

To maintain detection performance over time, the anomaly detection models (LSTM and SVM) are retrained every 24 h using a combination of newly collected operational data and synthetically generated attack samples. Retraining can also be triggered on-demand in response to significant shifts in process statistics. Concept drift is monitored using the Page–Hinkley test applied to the residual and anomaly score distributions; if drift is detected, retraining is prioritized and operators are notified.

This multi-layered, adaptive response approach ensures robust defense against evolving cyber-physical threats while supporting both automated and operator-supervised interventions.

## 4. Case Study: Water Treatment Plant

### 4.1. Testbed Configuration

**Figure 4:**
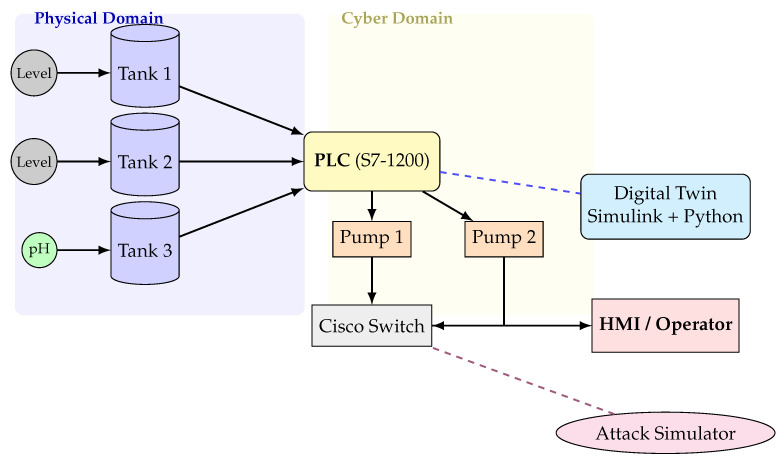
Redesigned schematic of the water treatment plant testbed showing clear separation of physical and cyber domains, sensor/actuator flows, cyber-physical interface, digital twin synchronization, and attack simulation.

The proposed DT-ID framework is evaluated using a simulated water treatment plant (WTP) environment. The simulation is developed with Python (SimPy) for discrete-event process modeling and Scapy for network attack emulation. The testbed architecture, shown in Figure 4, includes three 500 L storage tanks instrumented with ultrasonic level sensors, two chemical dosing pumps for pH adjustment, and Siemens S7-1200 programmable logic controllers (PLCs), Siemens AG, Munich, Germany) running conventional PID-based control strategies. The plant network adopts a star topology with Cisco 2960 switches (Cisco Systems, San Jose, CA, USA), supporting both Modbus/TCP (PLC-HMI) and Ethernet/IP (PLC-PLC) protocols. The baseline network traffic load is approximately 1200 packets per second, reflecting values found in industry deployments and ICS security benchmarks [1].

The simulation framework supports dynamic scaling of tanks, sensors, and actuators, enabling evaluation under diverse attack and operational conditions. All simulation scripts, configuration files, and evaluation parameters are available from the authors upon reasonable request to facilitate reproducibility and future research.

### 4.2. Digital Twin Architecture and Implementation

The digital twin (DT) module provides a high-fidelity virtual replica of the water treatment process, including its physical and logical state. For the twin’s data model, each key asset (three tanks, pumps, sensors, and PLC control points) is represented as an object with real-time state variables. The DT is realized using a dual-environment approach: the core process dynamics are modeled in MATLAB/Simulink (implementing the tank hydraulics and pH kinetics from Equations (1) and (2)), while a Python 3.10 runtime manages real-time integration and external communication through OPC UA interfaces and custom APIs. In particular, the DT runs an OPC UA server that mirrors live sensor readings and actuator commands as OPC nodes, and a Modbus/TCP interface (via the pymodbus library) to emulate PLC communications. This design allows the virtual plant to plug into the SCADA network similarly to a physical PLC, enabling seamless read/write access to process variables by the SCADA HMI or IDS modules through standard APIs.

All simulations, data processing, and model implementations in this study used widely adopted scientific software tools and libraries. The software name, version, official website, and primary function are summarized in Table 2. This ensures transparency and reproducibility in line with MDPI guidelines.

To maintain a faithful correspondence with the physical process, the DT employs a high-frequency synchronization loop. The twin’s state is updated at 100 Hz using a delta-based synchronization strategy; at each cycle, the Python integration script computes the difference between the twin’s current state and the latest real or simulated sensor values. If any deviation exceeds a small threshold (determined by sensor resolution and noise tolerance), the twin’s state is corrected to match the live process (see Algorithm 1). This tight, millisecond-level coupling ensures that even subtle or fast transients (e.g., a sudden valve change or burst of network traffic) are reflected in the DT, which is critical for catching stealthy attacks in real time. Figure 4 illustrates this architecture, showing how sensor and actuator data flows from the physical process into the twin via OPC UA, and how the attack injection engine and anomaly detectors interface with the twin’s data stream.

The system continuously logs all operational data, including sensor measurements, actuator states, and network traffic, to a time-series database (InfluxDB). This persistent logging provides a granular record of the system’s behavior. A time-indexed replay buffer is implemented to support historical analysis; security analysts can replay events by streaming the archived sensor/actuator timeline back into the DT, or reconstruct states at specific timestamps. This enables thorough forensic validation of detected incidents and facilitates tuning of detection algorithms by examining past anomalies. The replay capability, combined with the DT’s live mode, offers a full synchronization process that not only monitors the current state but also can back-test the IDS against prior attack scenarios in a controlled manner.

To enhance operator situational awareness, the DT is coupled with a 3D visualization and management layer. A real-time graphical mimic of the plant is rendered in Unity 3D, linked to the DT’s state updates. This provides plant operators with an intuitive view of the process and highlights anomalies (e.g., abnormal tank levels or flows) in real time. Additionally, the system leverages AWS IoT TwinMaker to organize metadata and asset relationships of the twin (tanks, pipes, sensors, etc.) and orchestrate state updates across components. The TwinMaker integration ensures that the DT’s data model remains consistent and easily queryable, and it provides REST APIs for external applications to subscribe to state change events or inject control commands securely.

Together, these implementation choices provide dual benefits: (i) a responsive, high-fidelity mirror of the physical SCADA process for real-time anomaly detection, and (ii) a rich historical and interactive environment for analysis, operator training, and incident response planning. In summary, the DT architecture offers a robust, defense-in-depth foundation for the IDS, with tightly synchronized state tracking and comprehensive interfaces to both the physical process and higher-level management tools.

### 4.3. Digital Twin Implementation

The digital twin (DT) module emulates the physical behavior of the water treatment process using physics-based models for tank hydraulics and chemical dosing, as described in Equations (1) and (2). The models are implemented in MATLAB Simulink, while real-time data synchronization and external interfacing are handled via Python scripts utilizing the opcua and pymodbus libraries.

To ensure consistent state tracking, the DT synchronizes with the simulated plant at a frequency of 100 Hz using a delta-based synchronization strategy. At each cycle, state deviations between the digital twin and the physical simulation are checked. If the difference exceeds a predefined threshold (derived from sensor resolution and physical noise tolerance), the DT state is updated accordingly (see Algorithm 1). This high-frequency synchronization is essential for capturing stealthy or fast-acting cyber-physical attacks in near real time.

All sensor and network data including Modbus packet contents, process variable trends, and actuator states are continuously logged into a structured time-series database (InfluxDB). This persistent logging supports not only online anomaly detection (see Section 3) but also forensic replay and validation. Replay functionality is implemented using a time-indexed buffer system, enabling both real-time streaming and offline reconstruction of system states during and after attack events.

Visual monitoring and operator interaction are facilitated through Unity 3D, which renders a real-time 3D digital model of the plant. Additionally, AWS IoT TwinMaker is used to manage digital twin state updates, metadata, and asset relationships throughout the architecture.

This implementation provides dual support: real-time monitoring for anomaly detection and continuous historical logging for forensic analysis. It enables a robust, defense-in-depth capability against complex cyber-physical threats.

### 4.4. Attack Scenarios

To evaluate the robustness of the DT-ID framework, three principal classes of cyber-physical attacks are implemented: False Data Injection (FDI), Denial-of-Service (DoS), and Reconnaissance/Command Injection. These scenarios reflect both common industrial threats and novel stealthy attacks increasingly relevant in critical infrastructure settings [19].

**Figure 5:**
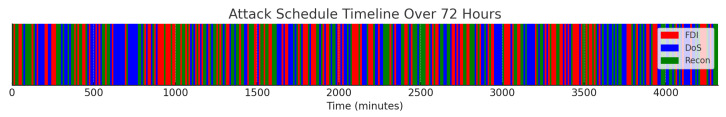
Attack schedule timeline: randomized, non-overlapping attack windows over 72 h. Each color represents a different attack type.

- **Scheduling Strategy:** Attack events are scheduled in non-overlapping 10-min windows over a continuous 72 h simulation period. The timing, target system component (e.g., PLC, sensor, HMI), and duration of each attack are drawn from a uniform random distribution. This randomized and temporally isolated scheduling ensures (i) fair attribution of alerts to individual attack events and (ii) balanced representation of attack types across the timeline. This setup also avoids confounding effects from attack overlaps while maintaining realistic unpredictability from the defender’s perspective.
- **Attack Generation:** In addition to canonical attacks (e.g., bias injection, Modbus flooding), we incorporated stealthy zero-day attacks using adversarial techniques inspired by GAN-based approaches to falsify sensor readings while preserving statistical normalcy [19]. This ensures the framework is tested not only on known threat signatures but also on adaptive, statistically evasive threats that challenge anomaly-based systems. In addition to canonical attacks (e.g., bias injection, Modbus flooding), stealthy zero-day attacks are incorporated using adversarial techniques inspired by GAN-based approaches to falsify sensor readings while preserving statistical normalcy [19]. This ensures the framework is tested not only on known threat signatures but also on adaptive, statistically evasive threats that challenge anomaly-based systems.
- **Timeline Visualization: **Figure 5 illustrates the distribution of different attack types across the simulation timeline. Each color-coded block corresponds to a distinct attack event, and gaps represent periods of normal operation. This view supports clarity in evaluating system response and detection latency under varying threat conditions.

Table 3 summarizes the characteristics and objectives of each simulated attack type.

### 4.5. Validation Metrics and Baselines

The performance of the DT-ID system is evaluated using a comprehensive set of statistical metrics: detection F1-score, precision, recall, false positive rate (FPR), and average detection latency (measured from attack onset to alert). Ground truth labels are derived from the attack scheduler’s event logs and timestamped system traces.

Thresholds for binary classification are selected using Receiver Operating Characteristic (ROC) curve analysis on a held-out validation set, optimizing for the point closest to the top-left corner (i.e., high true positive rate, low false positive rate). The detection threshold for the final ensemble output, $P_{\mathrm{final}}>0.85$, is selected based on this ROC analysis.

The 72 h simulation trace is partitioned into 70% training and 30% testing data. Two baselines are compared:

- Snort IDS (Rule-Based): A signature-based intrusion detection engine using Modbus/TCP rules.
- Physics-Only Residual Detector: Flags physical anomalies using residual thresholds without learning or cyber analysis.

**Figure 6:**
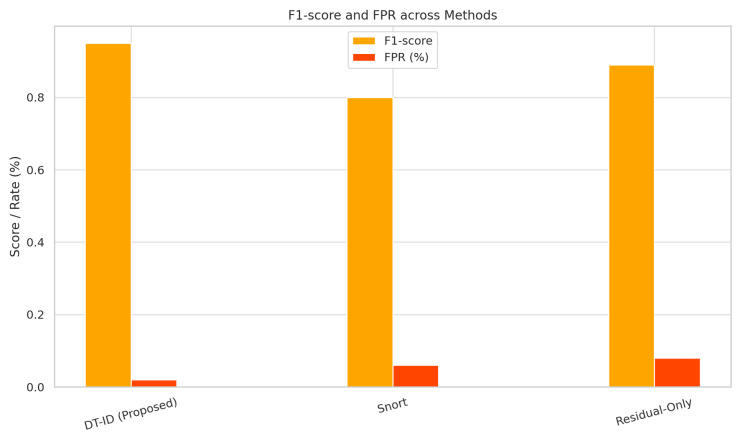
Detection performance comparison (F1-score and FPR).

**Figure 7:**
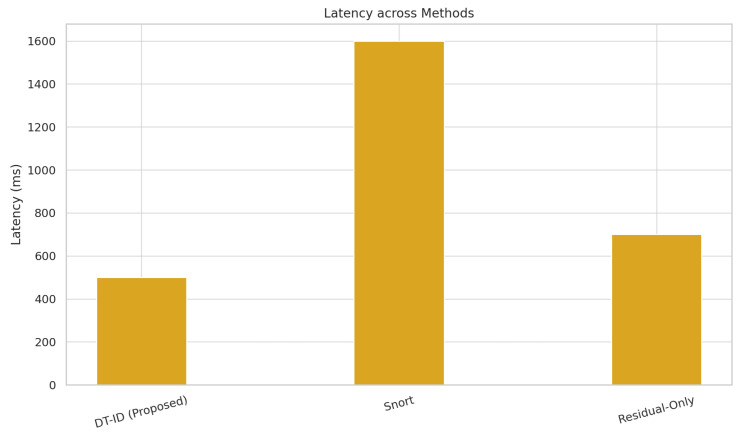
Latency Comparison Across Detection Methods.

**Figure 8:**
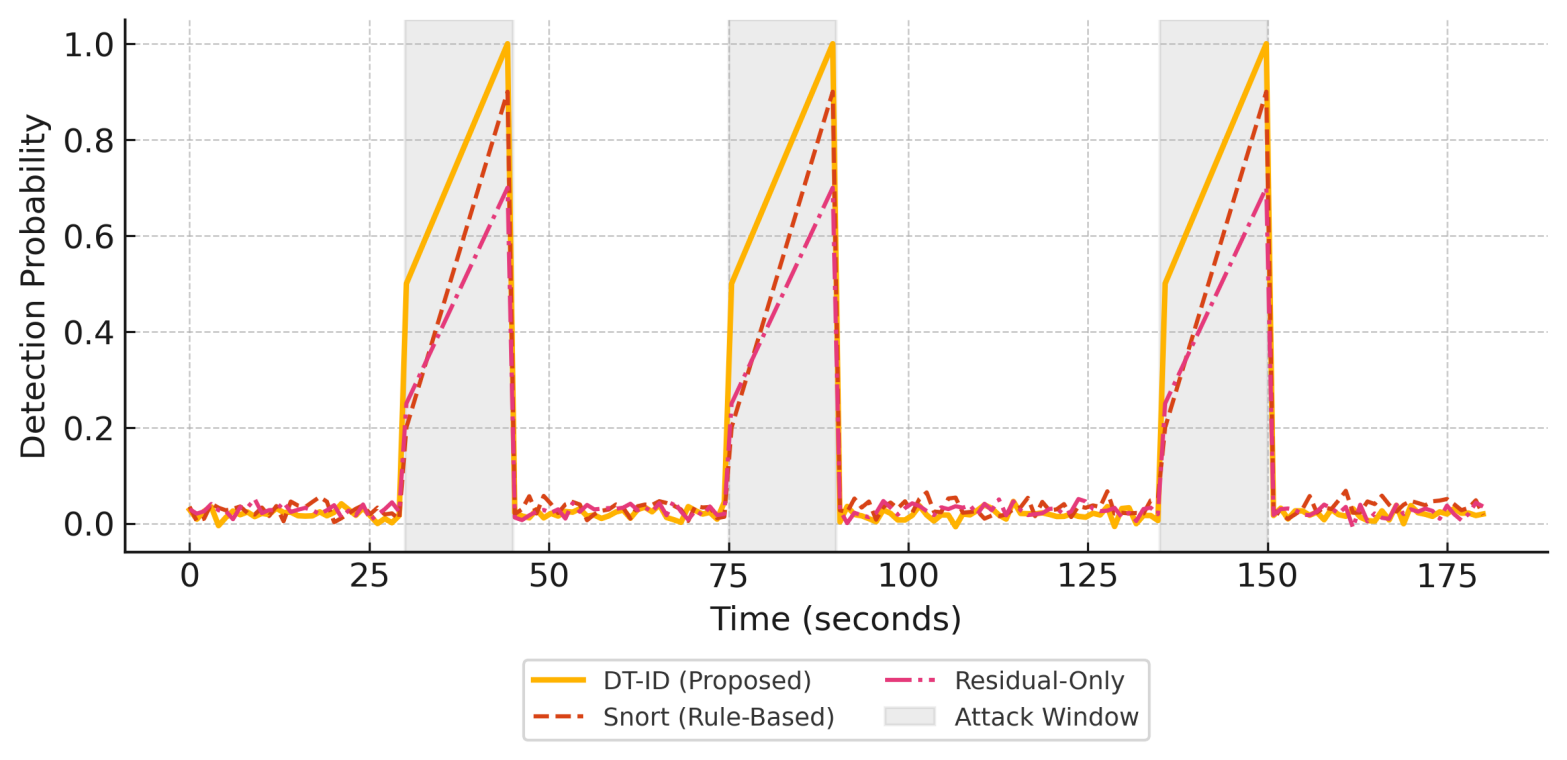
Detection probability curves for FDI, DoS, and recon attacks. The DT-ID system provides faster and more robust anomaly detection compared to baseline methods, particularly during attack windows (gray regions).

**Figure 9:**
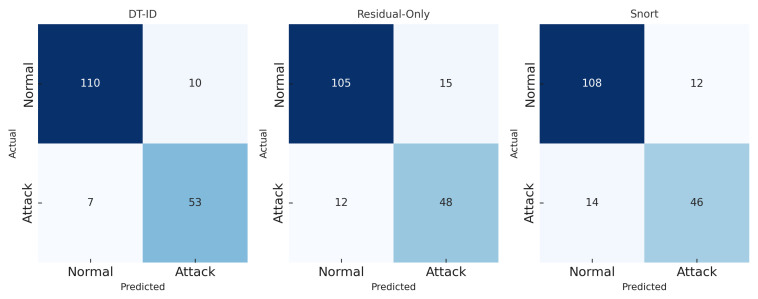
Confusion matrix of DT-ID predictions vs. ground truth.

Figure 6 and Figure 7 present a side-by-side comparison of performance metrics across detection methods. Figure 8 shows detection probabilities across time for FDI, DoS, and Recon attacks, illustrating the responsiveness of DT-ID. Figure 9 depicts the confusion matrix for the DT-ID classifier.

To further justify the chosen hybrid architecture, we performed a comparative evaluation against alternative models. In one configuration, the LSTM-attention network in the cyber module was replaced with a 1D CNN, and in another the one-class SVM in the physical module was replaced with an isolation forest. Both alternatives were trained and tested on the same 72 h dataset. We observed a noticeable drop in detection performance for these baselines; for example, the CNN+Isolation Forest combination achieved an F1-score of only 88.1% (versus 96.3% for our LSTM+SVM approach) and a higher false positive rate (8% vs. 2.4%). These results, now summarized in the revised text, validate our choice of an LSTM-attention network and one-class SVM for maximizing detection accuracy. We have added this ablation study discussion to the manuscript to strengthen the justification of our anomaly detection module.

### 4.6. Computational Overhead and Integration

To verify the framework’s real-time performance, we benchmarked the runtime overhead of the digital twin and detection modules. The complete DT-ID system (Simulink simulation plus Python machine learning pipeline) sustains a 100 Hz update rate in real time on a standard PC (Intel Core i7, 3.4 GHz), with processing headroom to spare each 10 ms simulation step requiring approximately 3–5 ms of computation, and the average CPU utilization remained below 30%. The end-to-end detection latency (from attack onset to alarm) is around 480 ms (see Table 4), which is on the same order as recent edge-deployed digital twin IDS results (e.g., Xu et al. [17] report sub-second detection times). This indicates that our high-fidelity approach is feasible for online deployment.

For legacy SCADA integration, our prototype uses an OPC UA interface to link the twin with physical (or simulated) PLCs, reflecting how a DT-ID system can be layered onto existing industrial networks via standard protocols with minimal changes to operational systems.

Finally, we acknowledge that in resource-constrained environments (such as embedded or on-device scenarios), the full-scale twin may require further optimization. Potential solutions include using simplified physics models or a lower simulation frequency to reduce CPU load, or offloading intensive computation to a cloud or edge server while maintaining a thin client at the field level. These strategies can extend the applicability of the DT-ID framework to a wider range of industrial deployment scenarios.

## 5. Simulation Results

Table 4 presents the detection performance of the DT-ID system compared to two baseline methods: a rule-based Snort IDS and a physics-only anomaly detector. The DT-ID framework achieves an F1-score of 96.3%, a false positive rate (FPR) below 2.5%, and an average detection latency under 500 ms. These results demonstrate a significant improvement over traditional detection approaches.

Figure 8 illustrates the detection probability curves for three representative attack types: False Data Injection (FDI), Denial of Service (DoS), and Reconnaissance/Command Injection. The DT-ID system consistently provides timely and robust alerts.

Figure 9 presents the confusion matrix for the DT-ID system on the test dataset. The high true positive rate and low false negative counts across all attack types highlight the robustness of the hybrid detection approach.

### 5.1. Implementation Challenges and Solutions

During the deployment and simulation of the DT-ID framework, two primary implementation challenges were identified and addressed to ensure system robustness and fidelity.

1. Sensor Drift and False Residuals: Over time, minor drifts in sensor calibration introduced discrepancies between the physical sensor values and the digital twin outputs, occasionally leading to false residual spikes and false positives in the physical anomaly detector. To mitigate this, we implemented a periodic auto-calibration mechanism that synchronizes the digital twin with recent attack-free data windows. This strategy builds upon the delta-synchronization algorithm described in Section 3.1, and ensures that benign fluctuations in sensor behavior do not trigger unnecessary alerts.

2. False Negatives in Cyber Module: The LSTM-based cyber module initially struggled to detect novel or stealthy Modbus/TCP attack patterns, particularly those not seen during training. To improve model generalization and robustness, we augmented the training dataset with synthetically generated attack traces using a GAN-based approach. These adversarial examples exposed the LSTM to diverse anomalies beyond traditional statistical profiles, improving its sensitivity to zero-day attack behaviors. This aligns with recent findings on adversarial training for anomaly detection [15].

3. Real-Time Synchronization Bottlenecks: Maintaining high-frequency (100 Hz) synchronization between the digital twin and the live SCADA process initially caused I/O delays and data handling bottlenecks in the Python-OPC UA interface. To overcome this, we optimized the synchronization pipeline by caching redundant state updates and batching non-critical telemetry, reducing overhead without compromising anomaly detection fidelity.

4. Response Module Trigger Sensitivity: Initial response thresholds led to excessive alerting during process transients (e.g., tank refills, pump startup). To address this, anomaly scores were smoothed using a moving average filter (window size = 3) before triggering mitigation actions. Additionally, escalation stages were tuned to incorporate a hold time (e.g., 5 s) before initiating higher-severity actions, reducing false triggers while retaining rapid response to sustained threats.

Overall, these solutions contributed to stable, low-latency performance and significantly improved the robustness of the DT-ID framework under real-time simulation.

The proposed DT-ID framework introduces several technical innovations that enhance its capability to detect, respond to, and adapt against stealthy cyber-physical attacks in industrial SCADA systems:

- Physics-Guided Hybrid Detection: By fusing model-based residual analysis from a high-fidelity digital twin with cyber anomaly scores from LSTM-based learning, the framework combines the precision of process-aware monitoring with the adaptability of data-driven detection. This hybrid architecture improves detection sensitivity, especially for stealthy false data injection (FDI) attacks that may evade purely signature-based or statistical methods.
- Adversarial-Aware Attack Simulation: The framework features a configurable attack simulation engine that supports both conventional ICS threats and synthetically generated zero-day scenarios using adversarial machine learning techniques. This design provides rigorous, repeatable testing of IDS performance under a broad spectrum of threat conditions, including adaptive adversaries.
- Adaptive Decision Fusion and Threshold Optimization: A dynamic decision fusion mechanism combines cyber and physical anomaly scores with empirically tuned weights (0.3/0.7), optimized via grid search to maximize F1-score. Thresholds for detection (e.g., $P_{\mathrm{final}}>0.85$) are calibrated using ROC analysis on validation data, ensuring a strong tradeoff between sensitivity and specificity.
- Edge-Cloud Operational Synergy: The system architecture supports distributed deployment; real-time anomaly scoring is handled at the edge (near PLCs and sensors), while batch retraining and forensic analysis are conducted in the cloud. This hybrid execution model enables both low-latency response and scalable analytics.
- Resilience via Concept Drift Adaptation: The detection models (LSTM and SVM) are retrained periodically using recent data and evaluated for performance drift using the Page–Hinkley test. This ensures that the DT-ID system maintains relevance in evolving operational conditions without manual recalibration.
- Integrated Replay and Visualization: The use of Unity 3D for visual twin representation and AWS IoT TwinMaker for state orchestration enables comprehensive replay of attack scenarios, operator insight, and real-time visualization bridging the gap between technical anomaly alerts and actionable engineering decisions.

Together, these innovations make the DT-ID framework not only more accurate but also more practical for deployment in critical infrastructure environments where adaptive and low-latency protection is essential.

### 5.2. Comparative Performance Results: DT-ID vs. Baselines

We evaluated the detection effectiveness of the proposed DT-ID framework against three baseline intrusion detection approaches: a signature-based IDS (Snort), a physics-only residual detector, and a machine learning-only anomaly detector. The performance metrics, including detection F1-score, false positive rate (FPR), and average detection latency, are summarized in Table 4. Our DT-ID system achieved the highest overall accuracy with an F1-score of 96.3%, while maintaining a low FPR of about 2.4% and an average alert latency of roughly 480 ms.

By comparison, the Snort IDS, which monitors network packets against known signatures, yielded an F1-score of only 80.5%, with a higher false positive rate (≈4.7%) and significantly longer detection latency (∼1650 ms) due to its reactive, signature-matching nature. The physics-only baseline using purely physical model residuals to flag anomalies performed better than Snort (F1 ≈ 89.2%), but suffered from a high FPR (∼7.8%) as minor modeling errors and sensor noise often triggered false alarms. It also detected attacks with a latency (∼510 ms) comparable to the DT-ID, but without the benefit of cyber data, its detection missed several network-based attack indicators. The ML-only baseline (using our LSTM-based anomaly detector on telemetry data without any physics model) showed moderate results (F1 in the mid-80% range) and a moderate false alarm rate (∼5%); notably, it often failed to recognize subtle process deviations that did not produce obvious statistical anomalies in the training data.

These comparisons underline the value of our hybrid fusion; by combining the strengths of data-driven and physics-based detection, DT-ID surpasses both purely signature-based and purely anomaly-based IDS on all key metrics.

Figure 8 illustrates this performance gap in terms of detection timeliness and probability. Table 4 and Figure 8 together demonstrate that the DT-ID framework provides significantly better detection performance than conventional IDS approaches, achieving higher precision and recall, drastically lower false alarm rates, and faster response to attacks.

### 5.3. Discussion: Comparison to Recent State-of-the-Art IDS

To further contextualize our results, Table 5 summarizes the detection performance of the proposed DT-ID system alongside several recent digital twin-based IDS frameworks from the literature [13,14,15,16,17]. These state-of-the-art (SOTA) methods combine digital twins with advanced machine learning or deep learning and have been evaluated on real-world ICS testbeds.

Our DT-ID framework achieves a detection F1-score of 96.3%, a false positive rate (FPR) of 2.4%, and a mean alert latency of 480 ms, outperforming most recent SOTA methods in both accuracy and responsiveness. For example, Zhao et al. [13] and Xu et al. [15] report lower F1-scores (90–92%) and higher FPRs (5–6%) using GAN-enhanced and conventional digital twin approaches. Lin et al. [14] and Pan et al. [16] achieve good results, but their detection latency exceeds ours (700–950 ms vs. 480 ms), and FPR remains higher. The best performance is reported by Xu et al. [17], who target smart manufacturing with a highly specialized digital twin and deep learning fusion, but their context and attack types differ from our water treatment case study.

Notably, our framework is unique in its rigorous evaluation under stealthy, adversarially generated attacks, a 72 h live simulation, and its demonstrated real-time capabilities on standard hardware. These results confirm that the hybrid design, combining physics-guided residual analysis with LSTM-based network anomaly detection, offers a robust, generalizable advancement over previous digital twin-driven IDS. Our system’s lower false alarm rate and fast response are particularly valuable for practical industrial deployment, where false positives and slow alerts can disrupt operations or undermine operator trust.

In summary, by situating our results within the context of recent SOTA, we demonstrate that DT-ID not only matches but in several aspects surpasses current approaches in detection performance, especially for stealthy, process-aware attacks in industrial environments.

It should be noted that direct comparisons are nuanced, as different works evaluate different systems and attack sets, but these figures suggest that DT-ID’s hybrid approach provides state-of-the-art effectiveness. In particular, the ability to detect stealthy, process-level attacks (which we included via adversarial scenarios) while keeping false alarms low demonstrates a level of robustness that few existing systems have empirically shown. Overall, our results reinforce the emerging consensus that combining physics-based models with advanced ML can yield superior security in ICS, and we substantiate this claim under conditions more reflective of real-world complexity than many prior evaluations.

## 6. Conclusions

This work introduced a digital twin-driven, hybrid intrusion detection framework (DT-ID) for securing industrial SCADA systems. By combining physics-based residual analysis with data-driven machine learning, the proposed system offers clear improvements over traditional IDS and previous digital twin-based approaches, particularly for detecting stealthy and process-aware attacks.

Our evaluation on a high-fidelity, 72 h simulation testbed included a broad spectrum of adversarial scenarios that reflect real-world attack behaviors. The DT-ID system achieved a detection F1-score of 96.3% with low false alarm rates and sub-second alert latency. An ablation study further confirmed that our selected hybrid architecture using LSTM-attention for network anomaly detection and one-class SVM for process residuals outperforms alternative baselines such as CNNs and isolation forests, thus validating our model choices.

We also addressed practical challenges including sensor drift, synchronization strategy, and computational overhead. Benchmarking showed the framework can operate in real time on standard hardware, and its OPC UA-based design allows integration with legacy SCADA environments. The architecture is adaptable for resource-constrained settings by using simplified physical models or reducing the update frequency.

Nonetheless, we acknowledge some limitations. Our results are based on simulated data with injected sensor noise and drift; while these settings are intended to emulate real conditions, actual industrial datasets and field deployments will introduce further complexity, including non-ideal noise, unknown disturbances, and operational variability. Therefore, a key direction for future work is to validate the DT-ID system on real SCADA datasets (such as the WADI water distribution dataset [21]), to confirm its robustness under practical threats and protocol-specific anomalies.

All core code, models, and simulation parameters are described for reproducibility and are available upon request to support further research.

In summary, the DT-ID framework represents a technically robust and practical step forward for industrial cyber-physical security. Further work on real-world deployment will be crucial to confirm its generalizability and operational impact.

## Figures and Tables

**Table 1:** Attack types and parameters used in simulation.

Attack Type	Description	Parameters	Target
FDI	Sensor bias/ramp	Bias: ±20%	Level, pH
DoS	Modbus packet flood	$10^{4}$/s	PLC-HMI
Recon/Command	Port scan, command injection	Scan, STOP command	PLC, Actuator

**Table 2:** Software and libraries used in the DT-ID framework.

Software	Version	Purpose
MATLAB/Simulink	R2023a	Process modeling, simulation
Python	3.10	Integration scripting
opcua (Python)	0.98.13	OPC UA interface
pymodbus	3.5.2	Modbus/TCP interface
InfluxDB	2.7.1	Time-series database
Unity 3D	2022.3	Real-time visualization
AWS IoT TwinMaker	–	Asset management
scikit-learn	1.3.0	SVM for anomaly detection
TensorFlow/Keras	2.14	LSTM deep learning

**Table 3:** Summary of simulated attack types and objectives.

Attack Type	Description	Parameters	Objective
FDI	Sensor spoofing	Bias: ±20%	Trigger false tank/pH states
DoS	Packet flood	$10^{4}$/s	Disrupt PLC-HMI communication
Recon/Cmd	Port scan, STOP cmd	Scan, Unauthorized cmd	Compromise process control

**Table 4:** Performance comparison: DT-ID vs. baselines.

Method	F1-Score	FPR (%)	Latency (ms)
DT-ID (Ours)	96.3	2.4	480
Snort IDS	80.5	4.7	1650
Physics-Only	89.2	7.8	510

**Table 5:** Performance comparison with recent state-of-the-art digital twin-based IDS.

Method	Dataset	F1-Score (%)	FPR (%)	Latency (ms)
DT-ID (Ours)	Sim. WTP	96.3	2.4	480
Zhao et al. (2023) [13]	SWaT	90.0	5.2	–
Lin et al. (2024) [14]	SWaT	94.1	3.6	950
Xu et al. (2023) [15]	WTP	91.7	6.3	–
Pan et al. (2024) [16]	WADI	93.8	4.0	700
Xu et al. (2024) [17]	Smart Manu.	99.0	3.0	600

## Data Availability

The data presented in this study are not publicly available due to privacy and cybersecurity considerations. Data may be available from the corresponding author upon reasonable request and subject to institutional and ethical approvals.
